# Prognostic Value of Programmed Cell Death Ligand-1 Expression in Nasopharyngeal Carcinoma: A Meta-Analysis of 1,315 Patients

**DOI:** 10.3389/fonc.2019.01111

**Published:** 2019-10-25

**Authors:** Xiaofeng Liu, Chunguang Shan, Yingluan Song, Juan Du

**Affiliations:** ^1^Department of Otolaryngology, Children's Hospital of Hebei Province, Shijiazhuang, China; ^2^Department of Otorhinolaryngology Head and Neck Surgery, The Second Hospital of Hebei Medical University, Shijiazhuang, China; ^3^Department of Neurothoracic Surgery, Children's Hospital of Hebei Province, Shijiazhuang, China

**Keywords:** PD-L1, meta-analysis, prognosis, nasopharyngeal carcinoma, clinical use

## Abstract

**Background:** The prognostic value of programmed cell death ligand-1 (PD-L1) in patients with nasopharyngeal carcinoma (NPC) remains controversial. Therefore, we conducted this meta-analysis to understand the role of PD-L1 in NPC.

**Method:** We searched PubMed, Embase, Web of Science, and Cochrane Library up to April 2019. We determined the pooled hazard ratio (HR) and 95% confidence intervals (CIs) to assess the relationship between PD-L1 and various survival outcomes. Begg's funnel plot was used to assess any publication bias.

**Results:** Eleven studies involving 1,315 patients were included in this meta-analysis. For overall survival (OS), the HR was 1.48 and 95% CI was 1.00–2.18 (*p* = 0.049). For disease-free survival (DFS), the HR was 1.51 and 95% CI was 0.85–2.69 (*p* = 0.162). For distant metastasis-free survival (DMFS), the HR was 1.75 and 95% CI was 0.64–4.79 (*p* = 0.277). For local recurrence-free survival (LRFS), the HR was 0.67 and 95% CI was 0.06–8.16 (*p* = 0.756). The results of prognosis of PD-L1 and OS were more significant after sensitivity analysis. The pooled odds ratio indicated that PD-L1 expression was not associated with T stage, N stage, M stage, overall stage, sex, age, smoking, or alcohol intake. No publication bias was found.

**Conclusion:** Our meta-analysis showed that PD-L1 overexpression in NPC was associated with a poor OS and may be useful as a novel prognostic factor for NPC.

## Introduction

Nasopharyngeal carcinoma (NPC) originates from the nasopharynx epithelium. NPC is rare compared with other types of cancer; moreover, its geographical distribution presents a unique pattern (1): it is a highly common type of head and neck cancer in the eastern and southeastern parts of Asia. NPC tends to metastasize to distant sites in the head and neck, and about 70% of the patients with NPC present with stage III or IV disease at the time of initial diagnosis (2). Radiotherapy (RT) is primarily used to treat NPC; however, concurrent chemoradiotherapy (CCRT) is recommended as the standard treatment for locoregionally advanced NPC (3). Although the 5-year survival rate of patients with NPC is ~60–70%, NPC management remains challenging because of locoregional failure, recurrence, and distant metastasis after primary treatment (4). Currently, several prognostic parameters are used for NPC management, including age and sex (5). However, these parameters lack sensitivity or specificity in some patients with NPC, and are, therefore, insufficient for predicting survival outcomes. Thus, the identification of novel and efficient prognostic markers is highly important in NPC treatment.

Programmed cell death ligand-1 (PD-L1), also known as B7-H1 or CD274, was cloned in 1999 (6). PD-L1 belongs to the B7/CD28 co-stimulator superfamily and is highly expressed in tumor-associated antigen-presenting cells (APCs), dendritic cells (7), macrophages (8), T cells (8), and various types of cancer cells (9, 10). PD-L1 along with programmed cell death 1 (PD-1; CD279), which is an inhibitory receptor expressed by tumor-infiltrating lymphocytes, can induce T cells apoptosis and inhibit the proliferation of immune cells (11): PD-1 and PD-L1 constitute the immune checkpoint that promotes tumor immune evasion (12). Using immunohistochemistry (IHC), PD-L1 overexpression was shown to be associated with a poor prognosis across multiple tumor types (10, 13), including non-small cell lung cancer (14), hepatocellular carcinoma (15), colorectal cancer (16), and renal cell carcinoma (17). Several studies also demonstrated the relationship between PD-L1 expression and prognosis of patients with NPC; however, results were controversial (18–23). Therefore, we conducted a meta-analysis to assess the impact of PD-L1 on the prognosis of patients with NPC.

## Materials and Methods

### Literature Search

Eligible studies were identified by searching PubMed, Embase, Web of Science, and Cochrane Library up to April 2019. The following search terms were used: “Programmed Cell Death Ligand 1” or “Programmed Death Ligand 1” or “PDL1” or “B7-H1” or “CD274” or “Programmed Cell Death 1” or “Programmed Death 1” or “PD-1” or “CD279” and “nasopharyngeal carcinoma” or “nasopharyngeal cancer” or “NPC.” References in the retrieved articles were also manually searched for additional studies.

### Inclusion and Exclusion Criteria

Eligible studies were identified according to the following inclusion criteria: (1) the NPC cases were pathologically confirmed; (2) PD-L1 expression was detected in NPC by using IHC; (3) studies provided the association between PD-L1 and survival outcomes and/or clinical characteristics; (4) studies provided the hazard ratio (HR) and 95% confidence interval (CI) for survival outcomes or sufficient information to calculate the HR and 95% CI in accordance with the methods by Parmar (24) and Tierney (25); (5) for studies (22, 26, 27) detected the expression of PD-L1 in both tumor cells (TCs) and immune cells (ICs), we only extracted the data of PD-L1 expression on TCs for analysis; and (6) original articles were published in English. The following were the exclusion criteria: (1) meeting abstracts, case reports, reviews, or letters; (2) duplicate studies; and (3) non-human studies.

### Data Extraction and Quality Assessment

Two authors extracted relevant data independently, and disagreements were settled by discussion. The following data were extracted: the name of the first author; the year of publication; country/region; sample size; age; tumor stage; treatment modality; study design; study duration; and HR and 95% CI related to PD-L1 expression. The quality of eligible studies was evaluated according to the Newcastle-Ottawa Scale (NOS) (28). The scale includes three categories: selection, comparability, and outcome assessment. The top score is 9 points, and studies with a score >6 are regarded as high-quality studies.

### Statistical Analysis

This meta-analysis was performed according to the Preferred Reporting Items for Systematic Review and Meta-Analysis (PRISMA) guidelines (29). Pooled HR and 95% CI were determined to assess the relationship between the PD-L1 and various survival outcomes. The pooled odds ratio (OR) and 95% CI were determined to study the correlation between PD-L1 and clinicopathological features. Statistical heterogeneity among all studies was assessed using the *I*^2^ and Chi-squared test. Heterogeneity was considered statistically significant when the *I*^2^ > 50% or *P* < 0.10. A random-effects model was used when significant heterogeneity was present; alternatively, a fixed-effects model was used. Sensitivity analysis and subgroup analysis were adopted to assess the heterogeneity and stability of the results. Begg's funnel plot was used to assess any publication bias (30). All statistical analyses were conducted with Stata version 12.0 statistical software (Stata Corporation, College Station, TX, US). A *P* < 0.05 was considered statistically significant.

## Results

### Literature Search

**Figure 1:**
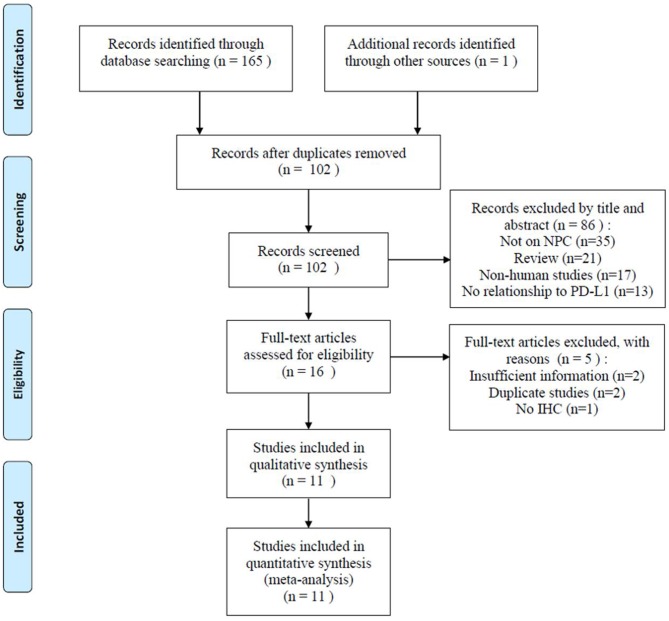
Flowchart of study selection.

A total of 166 related studies were initially retrieved. As shown in Figure 1, following the exclusion of 64 duplicate studies, 102 studies were screened. Eighty-six studies were excluded after title and/or abstract screening for the following reasons: not based on NPC (*n* = 35), reviews (*n* = 21), non-human studies (*n* = 17), and not related to PD-L1 (*n* = 13). Thereafter, the full text of 16 studies was assessed; five studies were excluded because of insufficient data (*n* = 2), duplicate studies from the same research group (*n* = 2), and no use of IHC (*n* = 1). Finally, 11 studies were included in this meta-analysis (18–23, 26, 27, 31–33).

### Study Characteristics

Baseline characteristics of the eligible studies are summarized in Table 1. The studies were published from 2016 to 2019 and were all conducted in Asia. Six studies were conducted in China (20–23, 32, 33), and one each were in Hong Kong (18), Philippines (19), Thailand (31), Taiwan (26), and Japan (27), respectively. Eight studies detected the expression of PD-L1 in TCs (18–23, 31–33) and 3 studies detected PD-L1 expression in both TCs and ICs (22, 26, 27). The data of PD-L1 expression of TCs were used for meta-analysis. Five studies recruited patients with both non-metastatic and metastatic NPC (19, 21, 26, 31, 33), 4 studies recruited patients with non-metastatic NPC (18, 23, 27, 32), and 2 studies did not provide the information on metastatic status (20, 22). Nine studies included non-treated NPC patients (18–20, 23, 26, 27, 31–33), one study recruited recurrent patients (21), and one study included previously-treated patients (22). In addition, 6 studies recruited patients with non-keratinizing histology type (WHO II) (19–21, 26, 31, 32), 2 studies included patients with both keratinizing and non-keratinizing histology types (27, 33), and 3 studies did not provide relevant data (18, 22, 23). The sample size ranged from 56 to 209 and the total number of patients was 1,315. There was one prospective study (23) and the remaining 10 studies were retrospective. The NOS scores ranged from 6 to 9 and the mean was 7.36; this indicated that all included studies were of high quality.

**Table 1:** Basic characteristics of eligible studies.

**References**	**Country** **/region**	**Sample** ** size**	**Sex (M/F)**	**Age**	**Tumor stage**	**Study period**	**Treatment** ** method**	**Detection method**	**Study** ** design**	**NOS score**	**Cell type**	**Metastatic status**	**Disease type**	**Histology type**
Lee et al. (18)	Hong Kong	104	85/19	53 (27–80)	I–IV	2005–2009	CCRT	IHC	Retrospective	7	TC	Non-metastatic	Non-treated	NA
Chang et al. (19)	Philippines	56	43/13	48.5 (19–71)	I–IV	2008–2011	CCRT	IHC	Retrospective	8	TC	Mixed	Non-treated	Non-keratinizing
Li et al. (20)	China	120	86/34	48 (17–69)	I–IV	2009–2012	CCRT, RT	IHC	Retrospective	7	TC	NA	Non-treated	Non-keratinizing
Zhou et al. (21)	China	132	106/26	46 (28–69)	II–III	2001–2013	CCRT, RT	IHC	Retrospective	7	TC	Mixed	Recurrent	Non-keratinizing
Zhu et al. (22)	China	209	150/59	52 (20–75)	I–IV	1991–2000	CCRT	IHC	Retrospective	8	TC, IC	NA	Previously-treated	NA
Cao et al. (23)	China	108	79/29	47 (16–68)	III–IV	2012–2014	CCRT	IHC	Prospective	9	TC	Non-metastatic	Non-treated	NA
Larbcharoensub et al. (31)	Thailand	114	77/37	51.6	I–IV	2007–2012	CCRT, RT	IHC	Retrospective	7	TC	Mixed	Non-treated	Non-keratinizing
Liu et al. (26)	Taiwan	208	146/62	49 (20–84)	I–IV	NA	CCRT	IHC	Retrospective	8	TC, IC	Mixed	Non-treated	Non-keratinizing
Ono et al. (27)	Japan	66	54/12	59.5 (13–85)	I–IV	2000–2015	CCRT	IHC	Retrospective	7	TC, IC	Non-metastatic	Non-treated	Mixed
Qu et al. (32)	China	96	72/24	NA	I–IV	NA	RT	IHC	Retrospective	6	TC	Non-metastatic	Non-treated	Non-keratinizing
Zhao et al. (33)	China	102	66/46	49 (23–76)	I–IV	2017–2018	CCRT	IHC	Retrospective	7	TC	Mixed	Non-treated	Mixed

*M, male; F, female; CCRT, concurrent chemoradiotherapy; RT, radiotherapy; IHC, Immunohistochemical staining; NOS, Newcastle-Ottawa Scale; TC, tumor cell; IC, immune cell; NA, not available*.

### Relationship Between PD-L1 Expression and Prognosis of NPC

**Figure 2:**
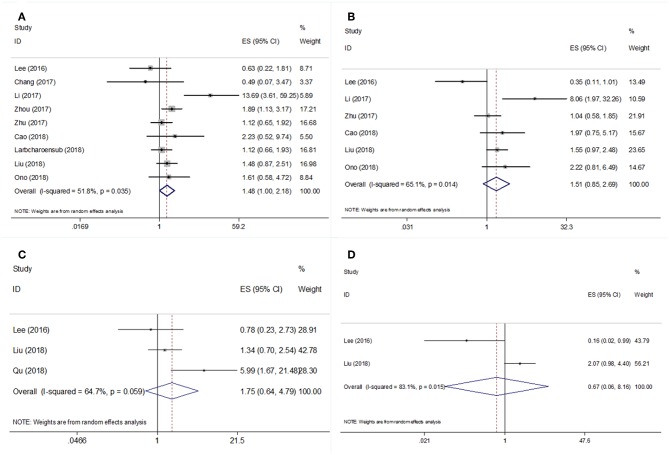
Forest plot diagrams of hazard ratios for correlations between PD-L1 expression and **(A)** OS, **(B)** DFS, **(C)** DMFS, and **(D)** LRFS.

Nine studies (18–23, 26, 27, 31) with 1,117 patients reported the prognostic value of PD-L1 regarding overall survival (OS). The results revealed that PD-L1 overexpression was associated with significantly poorer OS compared with PD-L1-negative tumors (HR = 1.48, 95% CI = 1.00–2.18, *p* = 0.049; Table 2 and Figure 2A). The pooled HR and 95% CI from six studies (18, 20, 22, 23, 26, 27) indicated that PD-L1 overexpression was not correlated with poor disease-free survival (DFS; HR = 1.51, 95% CI = 0.85–2.69, *p* = 0.162; Table 2 and Figure 2B). As shown in Table 2 and Figure 2, the pooled data also demonstrated that PD-L1 overexpression was not correlated with distant metastasis-free survival (DMFS; HR = 1.75, 95% CI = 0.64–4.79, *p* = 0.277) or local recurrence-free survival (LRFS; HR = 0.67, 95% CI = 0.06–8.16, *p* = 0.756).

**Table 2:** Meta-analysis of association between PD-L1 and OS, DFS, DMFS, LRFS in nasopharyngeal carcinoma.

**Subgroup**	**No. of** ** studies**	**No. of** ** patients**	**HR (95%CI)**	***p***	**Heterogeneity**		**Effects model**
					***I*^**2**^ (%)**	***P***	
OS	9	1,117	1.48 (1–2.18)	0.049	51.8	0.035	REM
**Tumor stage**							
I–IV	7	877	1.37 (0.84–2.24)	0.204	59	0.023	REM
II–III	1	132	1.89 (1.13–3.17)	0.016	–	–	–
III–IV	1	108	2.23 (0.52–9.60)	0.049	–	–	–
**Disease type**							
Non-treated	7	776	1.52 (0.86–2.70)	0.148	59.1	0.023	REM
Recurrent/ previously-treated	2	341	1.47 (1.01–2.14)	0.044	47	0.17	FEM
**Study design**							
Retrospective	8	1,009	1.44 (0.96–2.18)	0.081	56.8	0.023	REM
Prospective	1	108	2.23 (0.52–9.60)	0.283	–	–	–
DFS	6	815	1.51 (0.85–2.69)	0.162	65.1	0.014	REM
**Tumor stage**							
I–IV	5	707	1.45 (0.74–2.86)	0.283	71.1	0.008	REM
III–IV	1	108	1.97 (0.75–5.17)	0.172	–	–	–
**Treatment**							
Non-treated	5	606	1.70 (0.80–3.59)	0.165	68.6	0.013	REM
Recurrent/ previously-treated	1	209	1.04 (0.58–1.84)	0.902	–	–	–
DMFS	3	408	1.75 (0.64–4.79)	0.277	64.7	0.059	REM
LRFS	2	312	0.67 (0.06–8.16)	0.756	83.1	0.015	REM

*OS, overall survival; DFS, disease-free survival; DMFS, distant metastasis-free survival; LRFS, local recurrence-free survival; FEM, fixed-effects model; REM, random-effects model*.

### The Association of PD-L1 Expression With Clinicopathological Features

To explore the correlation between PD-L1 expression and clinicopathological characteristics of NPC, the pooled OR and 95% CI were determined. As shown in Table 3, the pooled data indicated that PD-L1 overexpression was not associated with T stage (OR = 1.25, 95% CI = 0.85–1.84, *p* = 0.261; random-effects model), N stage (OR = 0.98, 95% CI = 0.76–1.27, *p* = 0.885; fixed-effects model), M stage (OR = 0.79, 95% CI = 0.47–1.33, *p* = 0.374; fixed-effects model), overall stage (OR = 1.31, 95% CI = 0.95–1.81, *p* = 0.1; fixed-effects model), sex (OR = 1.20, 95% CI = 0.78–1.33, *p* = 0.871; fixed-effects model), age (OR = 1.06, 95% CI = 0.77–1.46, *p* = 0.726; fixed-effects model), smoking (OR = 0.81, 95% CI = 0.61–1.09, *p* = 0.162; fixed-effects model), or alcohol intake (OR = 0.72, 95% CI = 0.43–1.19, *p* = 0.196; fixed-effects model).

**Table 3:** The association between PD-L1 and clinical factors.

**Clinical factors**	**No. of** ** studies**	**No. of** ** patients**	**HR (95%CI)**	***p***	**Heterogeneity**		**Effects model**	**Begg's *p***
					***I*^**2**^ (%)**	***p***		
T stage (T3–T4 vs. T1–T2)	11	1,315	1.25 (0.85–1.84)	0.261	51.6	0.024	REM	0.938
Sex (male vs. female)	11	1,315	1.20 (0.78–1.33)	0.871	0	0.813	FEM	0.862
N stage (N2–N3 vs. N1–N0)	11	1,315	0.98 (0.76–1.27)	0.885	0	0.529	FEM	0.276
Smoking (yes vs. no)	8	906	0.81 (0.61–1.09)	0.162	0	0.45	FEM	0.536
Overall stage (III–IV vs. I–II)	7	913	1.31 (0.95–1.81)	0.1	0	0.782	FEM	0.881
M stage (M1 vs. M0)	6	785	0.79 (0.47–1.33)	0.374	0	0.78	FEM	0.260
Age (y) (>45 vs. ≤45)	6	767	1.06 (0.77–1.46)	0.726	45.4	0.103	FEM	0.851
Alcohol use (yes vs. no)	3	330	0.72 (0.43–1.19)	0.196	0	0.683	FEM	0.602

*FEM, fixed-effects model; REM, random-effects model*.

### Subgroup Analysis and Sensitivity Analysis

**Figure 3:**
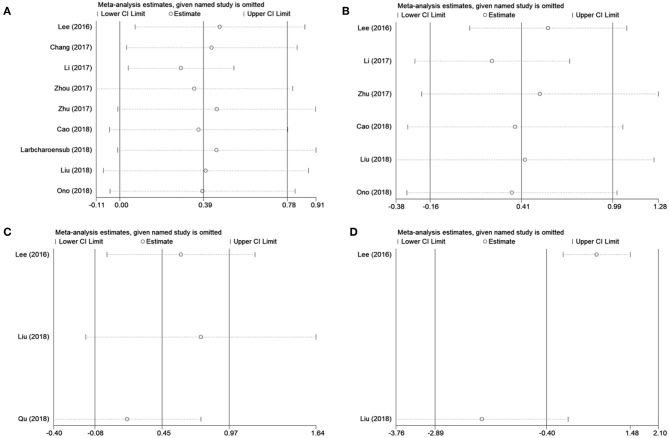
Sensitivity analysis for **(A)** OS, **(B)** DFS, **(C)** DMFS, and **(D)** LRFS.

Because several baseline characteristics varied among included studies, which may cause heterogeneity in meta-analysis. Therefore, subgroup analysis was conducted for OS and DFS. As shown in Table 2, PD-L1 expression was associated with poor OS in patients with stage II-III (*n* = 1, *p* = 0.016), stage III-IV (*n* = 1, *p* = 0.049), and recurrent/ previously-treated patients (*n* = 2, *p* = 0.044). However, PD-L1 was not significantly correlated to OS in both retrospective and prospective studies. Subgroup analysis also showed that PD-L1 remained a non-significant prognostic marker for DFS irrespective of tumor stage and disease type. Subgroup analysis was not performed for DMFS and LRFS because of the limited number of studies and uniformity among these studies. Sensitivity analysis by omitting one study in each turn was conducted to test the credibility of the prognostic value of PD-L1. As shown in Figure 3A, the results for OS were significantly altered when Li' s study (20) was excluded. Therefore, we re-analyzed the prognostic value of PD-L1 for OS after exclusion of Li's study (20), the results were HR = 1.33, 95% CI = 1.04–1.70, *p* = 0.022; *I*^2^ = 0, *P* = 0.513 (Figure S1). The heterogeneity in the analysis of PD-L1 and OS was significantly reduced after deletion of Li's study (*I*^2^ = 0, *P* = 0.513). No other individual study influenced the results for DFS, DMFS, and LRFS (Figure 3). The results of prognosis of PD-L1 and OS were more significant after sensitivity analysis, which suggested that PD-L1 could be a prognostic factor for OS in NPC patients.

### Publication Bias

**Figure 4:**
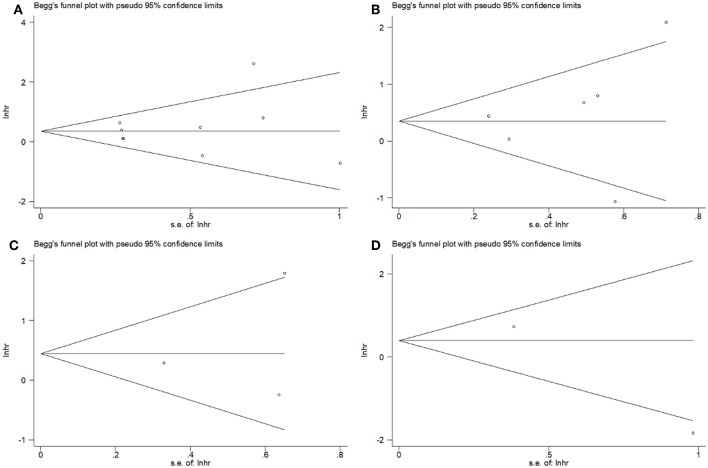
Funnel plots for publication bias of **(A)** OS, **(B)** DFS, **(C)** DMFS, and **(D)** LRFS.

Begg's tests showed that there was no publication bias in the eligible studies involving PD-L1 and OS (*p* = 0.602), DFS (*p* = 0.452), DMFS (*p* = 1), and LRFS (*p* = 0.317) (Figure 4). In addition, the funnel plots showed no publication bias for clinicopathological factors (Table 3).

## Discussion

Many studies demonstrated that PD-L1 plays an important role in tumor immune evasion (34, 35). The upregulation of PD-L1 inhibits T cell function and triggers immune evasion in cancer (36). In the tumor microenvironment, tumor-associated PD-L1 increases the apoptosis of T cells and leads to the growth of immunogenic tumors (36). PD-L1 is mainly expressed on the surface of tumor cells and tumor-associated APCs in various types of cancer including pancreatic cancer (37), ovarian cancer (38), thymoma (39), and colorectal cancer (40). PD-L1 and its receptor PD-1 constitute the PD-1/PD-L1 immune checkpoint signaling pathway (41). Checkpoint inhibition targets regulatory pathways in T cells to promote anti-tumor immune responses (12, 42). A recent phase II clinical trial including 44 patients revealed a promising activity of the PD-L1 antibody in NPC and a favorable 1-year OS rate (43). This study also suggested an association of PD-L1 expression with a higher response rate in patients with NPC (43).

To the best of our knowledge, this is the first meta-analysis to investigate the prognostic and clinicopathologic significance of PD-L1 expression in NPC. Survival data of 1,315 patients from 11 eligible studies were systematically analyzed. We found that PD-L1 overexpression was a significant prognostic factor for poor OS, whereas PD-L1 expression did not predict worse DFS, DMFS, or LRFS. Furthermore, PD-L1 expression was independent of T stage, N stage, M stage, overall stage, sex, smoking, age, or alcohol intake. Overall, this meta-analysis highlighted the potential of PD-L1 as a prognostic biomarker for poor OS in patients with NPC.

Previous meta-analyses exploring the prognostic value of PD-L1 in solid malignant tumors also indicated the unfavorable impact of PD-L1 on survival outcomes (10, 44, 45). A meta-analysis involving 13 studies showed that a high PD-L1 expression could predict a shorter OS (HR = 1.57, 95% CI = 1.09–2.27, *P* < 0.00001) and poorer DFS (HR = 2.07, 95% CI = 1.20–3.58, *P* = 0.009) in hepatocellular carcinoma (15). Another study also demonstrated a significant association of PD-L1 expression with a poor biochemical recurrence-free survival (BCR-FS) (HR = 1.78; 95% CI = 1.39 to 2.27; *p* < 0.00001) in prostate cancer (46). Wang et al. showed an association of PD-L1 expression with a poor OS in RCC (47). In addition, PD-L1 expression was found to be significantly associated with tumor stage, regional lymph node involvement, distant metastases, nuclear grade, and histologic tumor necrosis in RCC (47). The current meta-analysis showed the positive correlation of PD-L1 with OS, but not with DFS, DMFS, or LRFS. This may due to the limited sample size and the relatively short follow-up duration. Furthermore, we did not detect any association of PD-L1 with clinical factors in NPC. This requires further verification in future prospective studies.

There are several limitations in this study. First, all the eligible studies were conducted in Asia, which may reflect the high incidence of NPC in Asia. However, the results of this meta-analysis might be applied to patients in Asia. Second, although we selected eligible studies using uniform criteria, inter-study heterogeneity still exists in this meta-analysis. Therefore, subgroup analysis was performed to detect the source of heterogeneity. Third, the majority of the eligible studies were retrospective in design, which may compromise the validity of this study.

## Conclusions

In summary, this meta-analysis showed that PD-L1 overexpression in NPC was associated with poor OS and may be useful as a novel prognostic factor. Nevertheless, because of the aforementioned limitations, well-designed, large-scale, prospective clinical trials are required to verify the findings of this meta-analysis.

## Data Availability Statement

All datasets generated for this study are included in the article/Supplementary Material.

## Author Contributions

YS designed the study, performed the literature search and screening, performed the data analyses, and wrote the manuscript. XL and YS designed the study, retrieved the literature and data, analyzed the retrieved data, and participated in the writing of manuscript. CS and JD assisted in the designing of the study, performed the literature search and screening, assisted in the data analyses, and participated in the writing of manuscript. JD designed the study and supervised the study. Every author approved the final version of this study.

### Conflict of Interest

The authors declare that the research was conducted in the absence of any commercial or financial relationships that could be construed as a potential conflict of interest. The reviewer JM and the handling editor declared their shared affiliation.

## Supplementary Material

Figure S1 Meta-analysis of PD-L1 overexpression was associated with worse OS after deletion of Li's study.

## References

[1] Chua MLK Wee JTS Hui EP Chan ATC. Nasopharyngeal carcinoma. Lancet. (2016) 387:1012–24. 10.1016/S0140-6736(15)00055-0 26321262

[2] Chiesa F De Paoli F. Distant metastases from nasopharyngeal cancer. ORL J Otorhinolaryngol Relat Spec. (2001) 63:214–6. 10.1159/000055743 11408815

[3] Chen L Hu CS Chen XZ Hu GQ Cheng ZB Sun Y. Concurrent chemoradiotherapy plus adjuvant chemotherapy versus concurrent chemoradiotherapy alone in patients with locoregionally advanced nasopharyngeal carcinoma: a phase 3 multicentre randomised controlled trial. Lancet Oncol. (2012) 13:163–71. 10.1016/S1470-2045(11)70320-5 22154591

[4] Ma BBY Chan ATC. Recent perspectives in the role of chemotherapy in the management of advanced nasopharyngeal carcinoma. Cancer. (2005) 103:22–31. 10.1002/cncr.20768 15565580

[5] Sun R Qiu HZ Mai HQ Zhang Q Hong MH Li YX. Prognostic value and differences of the sixth and seventh editions of the UICC/AJCC staging systems in nasopharyngeal carcinoma. J Cancer Res Clin Oncol. (2013) 139:307–14. 10.1007/s00432-012-1333-9 23070122 PMC11824161

[6] Dong HD Zhu GF Tamada K Chen LP. B7-H1, a third member of the B7 family, co-stimulates T-cell proliferation and interleukin-10 secretion. Nat Med. (1999) 5:1365–9. 10.1038/70932 10581077

[7] Curiel TJ Wei S Dong HD Alvarez X Cheng P Mottram P. Blockade of B7-H1 improves myeloid dendritic cell-mediated antitumor immunity. Nat Med. (2003) 9:562–7. 10.1038/nm863 12704383

[8] Kuang DM Zhao QY Peng C Xu J Zhang JP Wu CY. Activated monocytes in peritumoral stroma of hepatocellular carcinoma foster immune privilege and disease progression through PD-L1. J Exp Med. (2009) 206:1327–37. 10.1084/jem.20082173 19451266 PMC2715058

[9] Pyo JS Kang G Kim JY. Prognostic role of PD-L1 in malignant solid tumors: a meta-analysis. Int J Biol Markers. (2017) 32:E68–74. 10.5301/jbm.5000225 27470134

[10] Wang QQ Liu F Liu L. Prognostic significance of PD-L1 in solid tumor: an updated meta-analysis. Medicine 96:e6369. 10.1097/MD.0000000000006369 28471952 PMC5419898

[11] Karwacz K Bricogne C Macdonald D Arce F Bennett CL Collins M. PD-L1 co-stimulation contributes to ligand-induced T cell receptor down-modulation on CD8^+^ T cells. EMBO Mol Med. (2011) 3:581–92. 10.1002/emmm.201100165 21739608 PMC3191120

[12] Chen J Jiang CC Jin L Zhang XD. Regulation of PD-L1: a novel role of pro-survival signalling in cancer. Ann Oncol. (2016) 27:409–16. 10.1093/annonc/mdv615 26681673

[13] Patel SP Kurzrock R. PD-L1 expression as a predictive biomarker in cancer immunotherapy. Mol Cancer Ther. (2015) 14:847–56. 10.1158/1535-7163.MCT-14-0983 25695955

[14] Hu XY Zhang W Hu Y Zhang Y Gong R Liang JY. A meta-analysis reveals prognostic role of programmed death ligand-1 in Asian patients with non-small cell lung cancer. J Huazhong Univ Sci Technol Med Sci. (2016) 36:313–20. 10.1007/s11596-016-1585-8 27376797

[15] Li JH Ma WJ Wang GG Jiang X Chen X Wu L. Clinicopathologic significance and prognostic value of programmed cell death ligand 1 (PD-L1) in patients with hepatocellular carcinoma: a meta-analysis. Front Immunol. (2018) 9:2077. 10.3389/fimmu.2018.02077 30254644 PMC6141709

[16] Shen Z Gu L Mao D Chen M Jin R. Clinicopathological and prognostic significance of PD-L1 expression in colorectal cancer: a systematic review and meta-analysis. World J Surg Oncol. (2019) 17:4. 10.1186/s12957-018-1544-x 30609938 PMC6320581

[17] Iacovelli R Nole F Verri E Renne G Paglino C Santoni M. Prognostic role of PD-L1 expression in renal cell carcinoma. A systematic review and meta-analysis. Target Oncol. (2016) 11:143–8. 10.1007/s11523-015-0392-7 26429561

[18] Lee VHF Lo AWI Leung CY Shek WH Kwong DLW Lam KO. Correlation of PD-L1 expression of tumor cells with survival outcomes after radical intensity-modulated radiation therapy for non-metastatic nasopharyngeal carcinoma. PLoS ONE. (2016) 11:e0157969. 10.1371/journal.pone.0157969 27341634 PMC4920427

[19] Chang AMV Chiosea SI Altman A Pagdanganan HA Ma CQ. Programmed death-ligand 1 expression, microsatellite instability, epstein-barr virus, and human papillomavirus in nasopharyngeal carcinomas of patients from the Philippines. Head Neck Pathol. (2017) 11:203–11. 10.1007/s12105-016-0765-y 27807760 PMC5429283

[20] Li YF Ding JW Liao LM Zhang ZL Liao SS Wu Y. Expression of programmed death ligand-1 predicts poor outcome in nasopharyngeal carcinoma. Mol Clin Oncol. (2017) 7:378–82. 10.3892/mco.2017.1318 28781814 PMC5530303

[21] Zhou Y Miao J Wu H Tang H Kuang J Zhou X. PD-1 and PD-L1 expression in 132 recurrent nasopharyngeal carcinoma: the correlation with anemia and outcomes. Oncotarget. (2017) 8:51210–23. 10.18632/oncotarget.17214 28881642 PMC5584243

[22] Zhu Q Cai MY Chen CL Hu H Lin HX Li M. Tumor cells PD-L1 expression as a favorable prognosis factor in nasopharyngeal carcinoma patients with pre-existing intratumor-infiltrating lymphocytes. Oncoimmunology. (2017) 6:e1312240. 10.1080/2162402X.2017.1312240 28638740 PMC5467992

[23] Cao C Wei Q Tang X Jia Y Sun X Li W. PD-1 and PD-L1 in locoregionally advanced nasopharyngeal carcinoma: a substudy of a randomized phase III trial. Head Neck. (2018) 41:1427–33. 10.1002/hed.25601 30582240

[24] Parmar MKB Torri V Stewart L. Extracting summary statistics to perform meta-analyses of the published literature for survival endpoints. Stat Med. (1998) 17:2815–34. 10.1002/(SICI)1097-0258(19981230)17:24<2815::AID-SIM110>3.0.CO;2-8 9921604

[25] Tierney JF Stewart LA Ghersi D Burdett S Sydes MR. Practical methods for incorporating summary time-to-event data into meta-analysis. Trials. (2007) 8:16. 10.1186/1745-6215-8-16 17555582 PMC1920534

[26] Liu YJ Tsang NM Hsueh C Yeh CJ Ueng SH Wang TH. Low PD-L1 expression strongly correlates with local recurrence in epstein-barr virus-positive nasopharyngeal carcinoma after radiation-based therapy. Cancers. (2018) 10:E374. 10.3390/cancers10100374 30304846 PMC6211078

[27] Ono T Azuma K Kawahara A Sasada T Matsuo N Kakuma T. Prognostic stratification of patients with nasopharyngeal carcinoma based on tumor immune microenvironment. Head Neck. (2018) 40:2007–19. 10.1002/hed.25189 29756253

[28] Wells GA Shea B O'connell D Peterson J Welch V Losos M The Newcastle-Ottawa Scale (NOS) for Assessing the Quality of Nonrandomised Studies in Meta-Analyses. (2009). Available online at: http://www.ohri.ca/programs/clinical_epidemiology/oxford.asp

[29] Moher D Liberati A Tetzlaff J Altman DG Grp P Preferred reporting items for systematic reviews and meta-analyses: the PRISMA statement. PLoS Med. (2009) 6:e1000097 10.1371/journal.pmed.1000097 19621072 PMC2707599

[30] Begg CB Mazumdar M Operating characteristics of a rank correlation test for publication bias. Biometrics. (1994) 50:1088–101. 10.2307/2533446 7786990

[31] Larbcharoensub N Mahaprom K Jiarpinitnun C Trachu N Tubthong N Pattaranutaporn P. Characterization of PD-L1 and PD-1 expression and CD8+tumor-infiltrating lymphocyte in Epstein-Barr virus-associated nasopharyngeal carcinoma. Am J Clin Oncol. (2018) 41:1204–10. 10.1097/COC.0000000000000449 29672367

[32] Qu Y Wang D Yang L Liu HY Cui W Che YQ. Expression and clinical significance of programmed death ligand 1 in nasopharyngeal carcinoma. Mol Clin Oncol. (2018) 9:75–81. 10.3892/mco.2018.1633 29977542 PMC6030993

[33] Zhao L Liao XY Hong GJ Zhuang YZ Fu KL Chen PQ. Mismatch repair status and high expression of PD-L1 in nasopharyngeal carcinoma. Cancer Manag Res. (2019) 11:1631–40. 10.2147/CMAR.S193878 30863173 PMC6388969

[34] Pardoll DM. The blockade of immune checkpoints in cancer immunotherapy. Nat Rev Cancer. (2012) 12:252–64. 10.1038/nrc3239 22437870 PMC4856023

[35] Tumeh PC Harview CL Yearley JH Shintaku IP Taylor EJ Robert L. PD-1 blockade induces responses by inhibiting adaptive immune resistance. Nature. (2014) 515:568–71. 10.1038/nature13954 25428505 PMC4246418

[36] Dong HD Chen LP. B7-H1 pathway and its role in the evasion of tumor immunity. J Mol Med. (2003) 81:281–7. 10.1007/s00109-003-0430-2 12721664

[37] Nomi T Sho M Akahori T Hamada K Kubo A Kanehiro H. Clinical significance and therapeutic potential of the programmed death-1 ligand/programmed death-1 pathway in human pancreatic cancer. Clin Cancer Res. (2007) 13:2151–7. 10.1158/1078-0432.CCR-06-2746 17404099

[38] Huang LJ Deng XF Chang F Wu XL Wu Y Diao QZ. Prognostic significance of programmed cell death ligand 1 expression in patients with ovarian carcinoma A systematic review and meta-analysis. Medicine. (2018) 97:e128858. 10.1097/MD.0000000000012858 30412078 PMC6221561

[39] Katsuya Y Fujita Y Horinouchi H Ohe Y Watanabe SI Tsuta K. Immunohistochemical status of PD-L1 in thymoma and thymic carcinoma. Lung Cancer. (2015) 88:154–9. 10.1016/j.lungcan.2015.03.003 25799277

[40] Ni X Sun X Wang D Chen Y Zhang Y Li W. The clinicopathological and prognostic value of programmed death-ligand 1 in colorectal cancer: a meta-analysis. Clin Transl Oncol. (2018) 21:674–86. 10.1007/s12094-018-1970-9 30392153

[41] Alsaab HO Sau S Alzhrani R Tatiparti K Bhise K Kashaw SK. PD-1 and PD-L1 checkpoint signaling inhibition for cancer immunotherapy: mechanism, combinations, and clinical outcome. Front Pharmacol. (2017) 8:561. 10.3389/fphar.2017.00561 28878676 PMC5572324

[42] Sharma P Allison JP. The future of immune checkpoint therapy. Science. (2015) 348:56–61. 10.1126/science.aaa8172 25838373

[43] Ma BBY Lim WT Goh BC Hui EP Lo KW Pettinger A. Antitumor activity of nivolumab in recurrent and metastatic nasopharyngeal carcinoma: an international, multicenter study of the mayo clinic phase 2 consortium (NCI-9742). J Clin Oncol. (2018) 36, 1412–8. 10.1200/JCO.2017.77.0388 29584545 PMC5941615

[44] Wu P Wu D Li LJ Chai Y Huang J. PD-L1 and survival in solid tumors: a meta-analysis. PLoS ONE. (2015) 10:e0131403. 10.1371/journal.pone.0131403 26114883 PMC4483169

[45] Shen X Zhao B Efficacy of PD-1 or PD-L1 inhibitors and PD-L1 expression status in cancer: meta-analysis. BMJ. (2018) 362:k3529 10.1136/bmj.k3529 30201790 PMC6129950

[46] Li Y Huang Q Zhou Y He M Chen J Gao Y. The clinicopathologic and prognostic significance of programmed cell death ligand 1 (PD-L1) expression in patients with prostate cancer: a systematic review and meta-analysis. Front Pharmacol. (2018) 9:1494. 10.3389/fphar.2018.01494 30733677 PMC6354218

[47] Wang Z Peng SH Xie H Guo LP Cai QL Shang ZQ. Prognostic and clinicopathological significance of PD-L1 in patients with renal cell carcinoma: a meta-analysis based on 1863 individuals. Clin Exp Med. (2018) 18:165–75. 10.1007/s10238-018-0488-3 29362922

