# Supplementary figures and images for: Prognostic Value of Programmed Cell Death Ligand-1 Expression in Nasopharyngeal Carcinoma: A Meta-Analysis of 1,315 Patients
Source: Front Oncol. 2019 Oct 25;9:1111. doi: 10.3389/fonc.2019.01111 (PMC6823255; doi:10.3389/fonc.2019.01111)

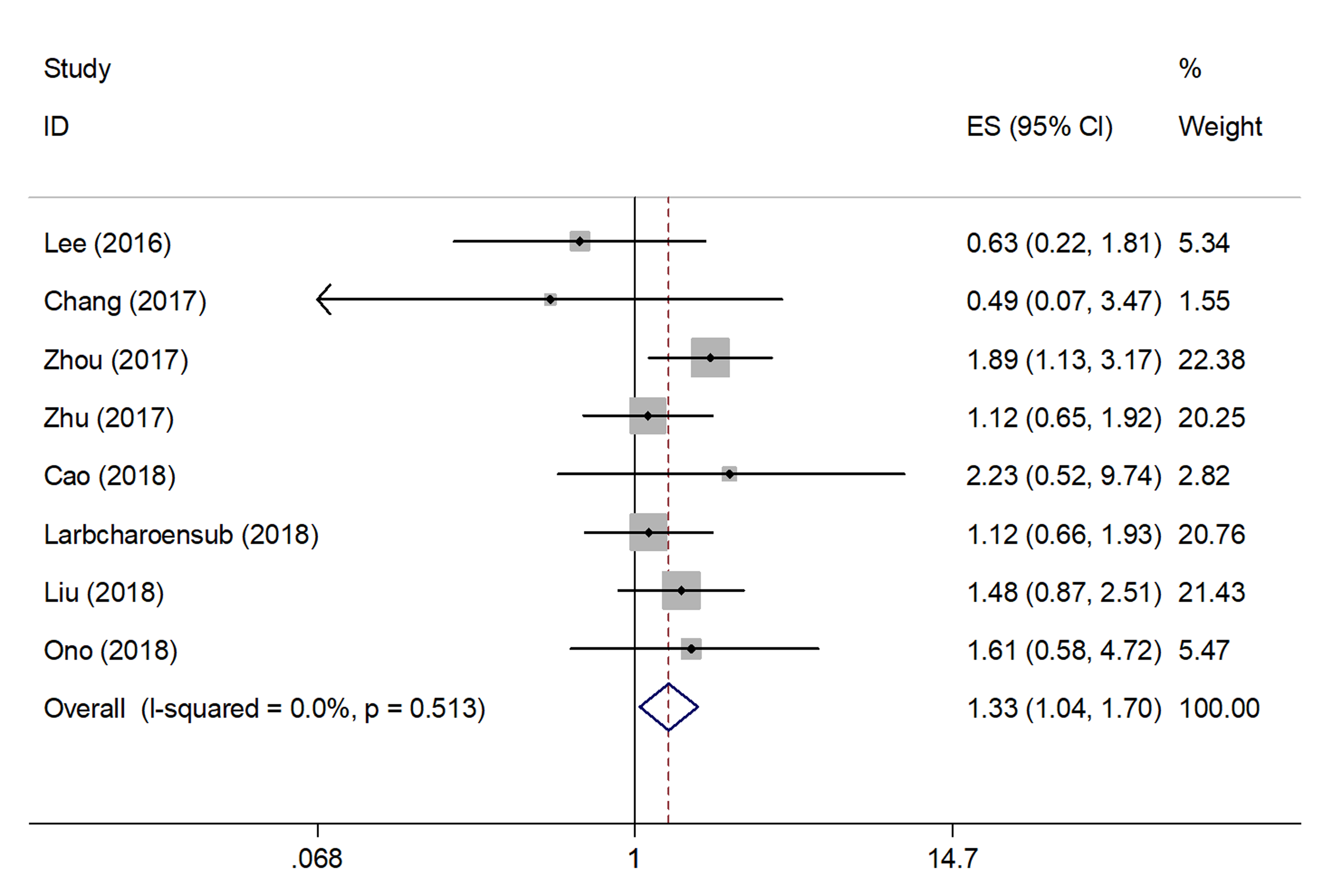

Supplement: Figure S1 — Meta-analysis of PD-L1 overexpression was associated with worse OS after deletion of Li's study. [file Image_1.tif]
